# Determination of the Transmittance Uniformity of Optical Filter Standard Reference Materials

**DOI:** 10.6028/jres.100.017

**Published:** 1995

**Authors:** J. C. Travis, N. K. Winchester, M. V. Smith

**Affiliations:** National Institute of Standards and Technology, Gaithersburg, MD 20899-0001

**Keywords:** charge-coupled device camera, filter certification, optical filters, spectrophotometry, standard reference materials, transmittance homogeneity

## Abstract

An instrument based on a scientific grade charge-coupled device (CCD) camera system is performance-qualified to evaluate the transmittance homogeneity of solid optical filter standard reference materials. Measurement results are presented for the new instrument, and compared, where appropriate, with an older, scanning instrument, for a variety of filters spanning transmittances down to 0.01. The new instrument is found to give comparable results with the older instrument, with reduced random uncertainty and improved information content.

## 1. Introduction

The Optical Filters program of the Analytical Chemistry Division of NIST produces standard reference material (SRM) optical filters, solutions, and purified chemicals, certified for their transmittance, transmittance density, or specific absorptivity at a variety of wavelengths throughout the ultraviolet (UV) and visible spectral regimes. The standards are used to verify the accuracy of transmittance and absorbance scales of UV/visible spectrophotometers. Although the solution and powder materials have many technical advantages, the most popular standards over the two decades of the program have been the solid optical filters mounted in metal frames designed to simulate the quartz cuvette sample holders accomodated by all laboratory-based UV/visible spectrophotometers. These filters offer the end user the ultimate simplicity in ease-of-use, though presenting NIST with special problems in their certification.

Unlike solution standards, the solid filters require individual certification of their transmittance using the Analytical Chemistry Division’s high accuracy spectrophotometer (HAS) [1], because neither the thickness of absorbing glass filters [2] nor the evaporative coating thickness for metal-on-quartz filters [3] may be adequately controlled for batch certification. Furthermore, the filters are certified over an area of 1 mm to 2 mm wide by 8 mm high, centered in the filter, as determined by the geometry and convergent beam of the HAS. Inasmuch as the beam geometry of end users may vary from that of the certifying instrument, a tolerance for transmittance homogeniety has been established for each class of optical filter standard over a 6 mm by 24 mm area, and individual filters have been accepted or rejected on the basis of a test using a scanning densitometer of NIST design and construction [4].

The maturity of scientific grade digital cameras based on charge coupled device (CCD) solid-state detector arrays [5, 6] has provided an opportunity to enhance the filter evaluation process. The linearity of these devices over a large dynamic range is sufficient to resolve the transmittance variations of interest, while the number of detection elements is adequate to examine multiple filters simultaneously, while still providing higher spatial resolution than the former instrument. The simultaneous acquisition of data over the area of interest of each filter minimizes the impact of temporal light source drift, and relaxes the requirements for absolute source stability.

The data presented herein represents the performance qualification of a new CCD camera-based instrument for homogeneity evaluation. The emphasis of the study is on the direct replacement of the original scanning instrument, with pixel averaging to yield a comparable sampling area, while still benefitting from improved measurement reproducibility and additional measurement areas on each filter. Future studies anticipate use of the system to identify sources of inhomogeneity and improve filter production quality, and to address the homogeneity issue for end user systems which illuminate narrower portions of the sample than the NIST instrument.

## 2. Experimental

### 2.1 Scanning Densitometer

The original scanning densitometer used to date for evaluating filter homogeneity contains a source, chopper, filter holder, detector and transfer lens in a 75 cm × 50 cm × 135 cm dark enclosure. The light source is a tritium activated phosphor button, chosen for its short term stability (12.3 year half-life), masked to an area of about 3 mm × 12 mm, and with a limited spectral bandwidth about an emission peak at 560 nm. A 20 cm focal length lens is employed to image the source on the filter under test, with a 3:1 image reduction for a final illuminated area of 1 mm × 4 mm. The light transmitted through the filter impinges on a diffuser-photomultiplier detector. The light is amplitude-modulated via an optical chopper located near the source, and the signal from the photomultiplier is processed by a lock-in amplifier before being digitized into a control computer.

The filter holder is mounted on a two-dimensional translation stage, with computer control via stepper motors, permitting the testing beam to pass through different positions on the filter. A matrix of nine positions is sampled, for three values each of the lateral position and height, with symmetry about the filter center which is one of the sampled positions. The nine positions are spaced on 1.75 mm centers horizontally and on 8 mm centers vertically. Thus, the outermost edges of the 1 mm by 4 mm beam sample an area 4.5 mm wide by 20 mm high, with a coverage of 40 %. The control computer prepares a report of the signal at each position, and the relative percent deviation of the signal at each position from that at the central position. The relative deviations are compared to the acceptance criteria for each filter type to determine the acceptance or rejection of a particular filter.

A single filter to be examined for homogeneity is mounted in one of the metal holders (simulating a 12.5 mm square cuvette) in which SRMs 930 and 1930 are distributed. The scanning densitometer takes 5 min to complete the scan of the single filter. Filters which clearly exceed the tolerance for inhomogeneity for a given transmittance level are removed from the holder and discarded. Marginal filters are often rerun to check the reproducibility of the test, and may be sent to the glass shop for repolishing. Filters which clearly have acceptable uniformity are passed along to the high accuracy spectrophotometer for further processing, and remain in the metal filter holder.

### 2.2 CCD Camera

The camera system is based on a Princeton Instruments TE/CCD-512TKUV scientific grade camera.^1^ Such cameras are characterized by careful hand selection of the detector array for quality and low defect incidence, digital signal processing with high dynamic range, and available cooling for the reduction of dark current derived noise. The detector array in the NIST camera has 512 rows and 512 columns of 27 *μ*m × 27 *μ*m detector elements, or pixels, covering a square area 13.82 mm on a side. The camera is cooled via a four stage Peltier effect device, and temperature controlled to an operating temperature of about −50 °C, with a control stability of 60.01 °C.

The dynamic range of the camera is limited by the 16 bit analog to digital (A/D) converter to one part in 65 000 (for a single exposure), and is further limited by other considerations. The camera exhibits a zero offset, or “dark” background, of approximately 600 “counts” (where one count is equivalent to the least significant bit of the A/D converter, for a maximum signal of 65 000 counts per exposure). The majority of this background does not represent “dark current,” but is a fixed offset required for electronic reasons. The camera software accomodates automatic subtraction of this offset background to restore measurement linearity. However, this nominally constant background exhibits a pixel-to-pixel estimated standard uncertainty of about 5 to 6 counts, which may arise from a combination of dark current noise, fixed pixel-to-pixel electronic effects, and electronic read noise.

The 600 count background subtraction amounts to very little loss of dynamic range, since 600 counts out of 65 000 is an insignificant fraction. However, images derived from data with maximum signal exceeding 30 000 counts have shown unacceptable distortions, perhaps due to “blooming”^2^ or other inter-pixel interactions, such that a practical limit of 30 000 counts, or about 15 bits of the A/D converter, is employed.

CCD arrays feature a fixed pixel-to-pixel variability in sensitivity (often termed “fixed pattern offset”) on the order of 1 %. For this reason, the camera accomodates “flatfield” correction as well as background subtraction. Flatfield correction is achieved by illuminating the detector with a uniform light source, and storing the image file as a record of the relative pixel-to-pixel sensitivity. The software accomodates dividing the current image by the stored flatfield image on a pixel-wise, shot-to-shot basis, along with subtraction of the background.

The camera system contains the detector head, a controller, a water-recirculator, and a computer and software for controlling the data acquisition and processing. Operating software is available for both the Windows and DOS environments. The menu-driven software operating under DOS was used for the fixed, repetitive protocol of optical filter testing.

### 2.3 Optical Configuration

For back illumination of a matrix of optical filters for simultaneous examination by the CCD camera, a Gordon Instruments photographic work station was obtained, containing a built-in 12.5 cm by 12.5 cm moderately uniform incandescent light source and an upright camera stand. In spite of the broad spectral bandwidth of the incandescent light source, the optical bandwidth detected by the camera is restricted to a 10 nm bandpass centered on the Hg wavelength at 546.1 nm by a 5 cm diameter interference filter positioned in the optical train between the camera shutter and the lens mount.

**Fig. 1:**
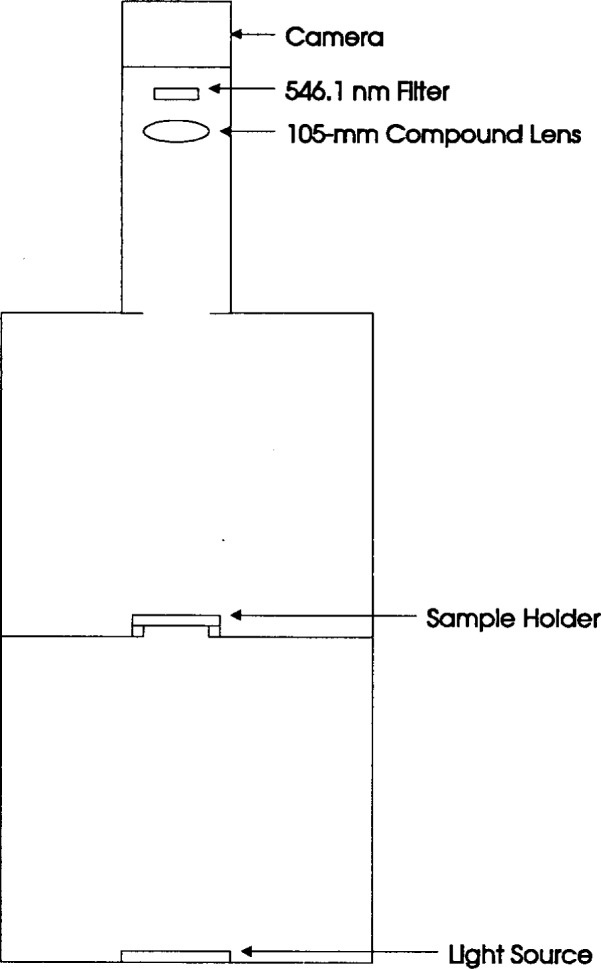
Block diagram of the camera-based system for determining the homogeneity of optical filter standard reference materials.

The extended light source is the bottom element of a vertically oriented optical system which has evolved into the configuration illustrated in Fig. 1. The central chamber, containing the filter holder at the bottom, is a light-tight 50 cm wide × 50 cm high × 25 cm deep box constructed of 18 mm plywood, laminated on the outside and painted flat black on the inside, with a light-tight sliding door to enable filter manipulation. This chamber was originally designed to constitute the entire geometry, with a 7.5 cm square hole in the bottom positioned directly over the extended source, and the camera mounted directly to the hole in the top. The top and bottom chambers are presently skeletal, with light protection provided by black felt, but will be replaced with permanent light-tight structures.

The top chamber was added to distance the camera, in order to reduce the variation of the viewing angle over the 5 mm by 20 mm area being examined for each filter. Preliminary results with a shorter focal length system revealed vignetting by the edges of the filter holder, exaggerated by multiple internal reflections and off-normal viewing, as well as apparent transmittance change along the long axis of a filter resulting from angular change confounded with multiple internal reflections. The 40 cm extension to the camera mount has reduced these effects to well under the acceptable margin of uncertainty. The multiple filter holder is mounted to the bottom of the box, for a distance of about 90 cm separating the filters from the detector array.

Though represented as a simple lens in Fig. 1, a 105 mm effective focal length compound “Macro” lens for a 35 mm camera (Nikon, AF Micro-Nikkor 105mm f/2.8 D) is employed to image the filters onto the detector array, with an image reduction of about 7:1. The filter holder contains eight filters in a plane, in a field of view of about 5.5 cm by 6 cm, at a distance of about 79 cm from the nearest surface of the compound lens.

Off-normal viewing also has some implication for flat-field correction, since the line-of-sight is shifted by refraction on passing through a glass or quartz filter of finite thickness. For the present conditions, a maximum “walk-off” of about 50 *μ*m at the filter surface corresponds to less than 10 *μ*m at the detector, which is still a significant fraction of a pixel. This factor becomes irrelevant for flat-field correction providing the source is sufficiently uniform across a small number of pixels.

In the original configuration, the light source was sufficiently close to the filter plane that imperfections and dust on either surface of the translucent cover plate of the extended light source were imaged onto the detector with partial blurring. The local source non-uniformity caused by such features, confounded with the offset in flat-field correction, left residual effects in the corrected image corresponding to the cover-plate imperfections.

The bottom chamber of Fig. 1 removes the light source an additional 45 cm from the filters, such that the cover plate is well out of focus in the final image, and successfully eliminates the source-derived artifacts. A second function of the lower stage is to reduce the total amount of light admitted to the sample chamber by reducing the solid angle of acceptance.

Eight filters are sandwiched between two pieces of 1.6 mm Al sheet, machined on the inner faces to “capture” the individual filters, and with 9 mm wide by 28 mm long oval apertures at each filter position on the top plate, to expose the area of interest of the filters while masking the edges. The bottom plate is more exposed, only suspending the ends of the filters, to minimize vignetting. A separate “blank” plate of thicker aluminum is provided with identical apertures to the top plate of the filter holder, and is used for obtaining the “flat-field” correction data. The blank and filter holder are alternately attached to an aluminum frame which is screwed to the floor of the central chamber. The positional reproducibility of the blank and filter holder is determined by the clearance between the attachment screws and the clearance holes, and seems to be within a pixel in the image, corresponding to about 150 *μ*m at the sample.

The present sample holder is not particularly user-friendly, requiring approximately 5 min to install the eight filters and a similar period to dismount them. It may prove advantageous to provide a holder suitable for filters which are already contained in the metal brackets in which the SRMs are sold, as they are for the other instrument.

### 2.4 Data Acquisition and Reduction

The camera and light source are turned on and allowed to equilibrate for at least 30 min before data collection is begun. The file used for background correction, that used for flatfield correction, and data files are all pixel by pixel averages of nine 0.5 s exposures. The background file is obtained with the light off or blocked, and has been found to be sufficiently stable and inconsequential that it is considered unnecessary to obtain a new file on a daily basis. The flatfield file is obtained at the beginning of each working day, and is retaken if the source is turned off and back on or if the intensity is changed. The flatfield data is taken with the “blank” mask in place, and the averaged background is automatically subtracted from the data at the end of each of the nine exposures, before averaging. For data collection with the sample holder in place, each of the nine exposures is followed by automatic background subtraction followed by automatic flatfield correction. The nine corrected exposures are then averaged, and the maximum resolution file may be saved on the computer to preserve a visual record. (The numerical treatments which follow result in intentional degradation of spatial resolution).

**Fig. 2:**
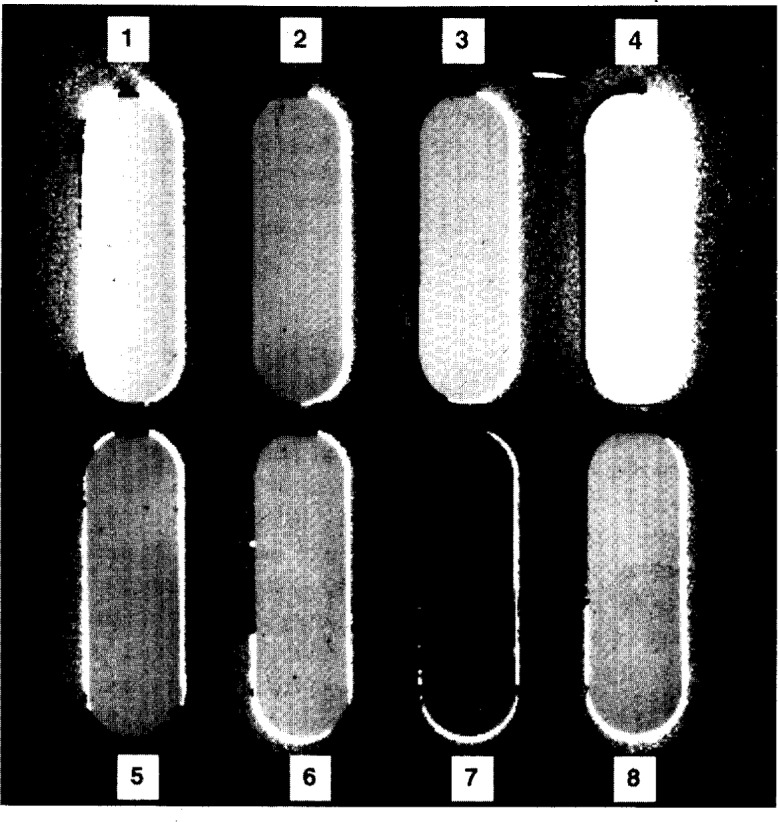
Image of eight nominal *T* = 20 % filters, including background correction and source intensity normalization of the image. Filter positions are designated as 1–8, from top left to bottom right, row-wise. The filters shown here are those listed in Table 2. The 8 bit greyscale was optimized for contrast using position 1 (filter 20-1), and filter 20-4, in position 4, is “offscale” and shown as white.

The camera system is normally employed with eight filters of the same nominal transmittance in the eight positions of the filter holder, for reasons discussed in Sec. 3.7. Figure 2 is an actual image of eight filters, in the holder, with overlaid numbers indicating the numbering system employed for the filter positions. The oval apertures primarily mask the corners of the filters, and about 1 mm about the edge. For experiments described herein using only two filters at each of several transmittance levels, the central four apertures of the blank and of the filter holder were blocked with black electrical tape. The unblocked outer positions of the filter holder were employed with the adjacent pair of positions on one edge holding a pair of filters of the same nominal transmittance and the pair of positions on the opposite edge holding a second pair of filters of the same nominal transmittance within the pair, and within a decade of that of the other pair. For instance, a pair of *T* = 1 % filters was run with a pair of *T* = 3 % filters, and a pair of *T* = 10 % filters was run with a pair of *T* = 30 % filters.

The optical image sufficiently underfills the detector array that only the center 402 of the 512 rows of pixels are employed. This matches the aspect ratio of the image to the available display space on the computer monitor, and facilitates convenient display of the entire image at once. It also speeds up many of the numerical operations by reducing the size of the data set. Further reduction of the data set is accomplished after storage of the image by software “binning” the image into a 256 × 201 array. This is accomplished in the furnished software, and represents averaging four adjacent pixels in two rows and two columns into a single “super pixel” in the final array. The binned array is then exported from the acquisition program as an ASCII file, for further processing by NIST-generated application-specific software.

**Fig. 3:**
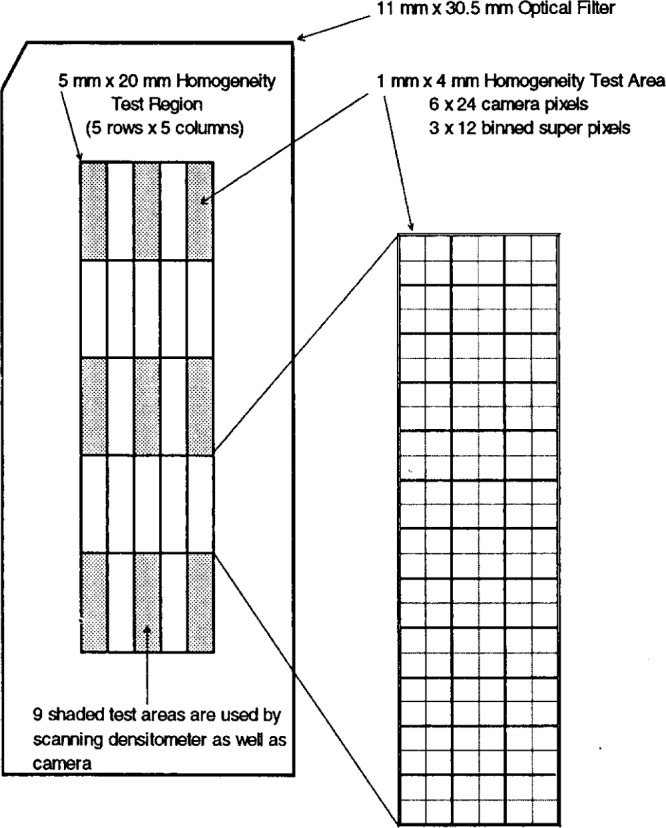
A “map” of the layout of a single filter, indicating the central region tested for homogeneity, the geometry of the 25 test areas used to determine homogeneity, and the relationship of a representative test area to CCD camera pixels and 2 × 2 “binned” super pixels employed in some of the computations. Nine of the 25 test areas, used in common with the scanning densitometer, are shaded for identification.

One such program prepares “reports” of the nominal transmittance and its variability over 25 nominal 1 mm by 4 mm sub-areas distributed in five rows and columns over an area of 5 mm by 20 mm centered in the face of each filter. A “map” of a filter, showing the central region over which homogeneity is determined, the 25 areas used to sample the transmittance, and the pixel and super pixel composition of each area, is given in Fig. 3. Of the 25 regions measured on each filter, the nine with odd row and column numbers correspond approximately to the nine regions measured on the scanning densitometer, and are grey-shaded in Fig. 3. However, exact correspondence has not been achieved because of the pixel “quantization” and the lack of iterative correction of the distances and magnification to achieve perfect registration of the two instruments.

Using “macro” command files with the camera software, the acquisition and automatic post-processing for either the flatfield file taken at the beginning of a work period or a “sample” run is approximately five min. Obviously, the nine 0.5 s exposures account for a relatively small portion of this time. Most of the time is consumed by computations and data handling. It may be possible to speed up the process with the optimization of data handling.

### 2.5 Optical Filter Samples

The filters chosen for the study were candidate filters for SRMs 930d and 1930, which had been rejected for excessive inhomogeneity by the scanning densitometer. Two filters each at nominal transmittances (*T*_nom_) of 1 %, 3 %, 10 %, and 30 % were measured in replicate on both the scanning densitometer and the CCD camera system. Eight *T*_nom_ = 20 % filters were also studied on each instrument, with careful identification and orientation of each filter. The unmounted filters are 11 mm by 30.5 mm on a side, and vary from 1.3 mm to 2.3 mm thick, to adjust the transmittance level for a particular base glass material. One corner of the filter is notched, for orientation. The notch is in the upper right corner as the filter is mounted in a spectrophotometer, and as viewed from the incident light direction.

## 3. Results and Discussion

The instrument being qualified is not used to report a certified value pertaining to a standard reference material. The sole function of the instrument is to reject filters which demonstrate transmittance variations which exceed the specifications for the particular SRM and nominal transmittance level. These specifications become a part of the estimated combined uncertainty for the certified transmittances (and derived transmittance densities) for the optical filter SRMs. Indeed, the scanning densitometer used to date for the purpose of homogeneity testing has never been rigorously qualified or tested to characterize the statistical uncertainty of the reported transmittance differences.

The new CCD camera system was inspired by the inability of the scanning densitometer to rapidly survey newly received materials, and by the dominance of random photon statistics (shot noise) for low transmittance filters. The camera is able to test unmounted glass filters in parallel, as well as evaporatively coated metal-on-quartz filters *before* the cover plate is optically contacted. Furthermore, the camera is able to operate at light levels well above those of the scanning densitometer, which is limited by the maximum specific activity of the tritium-activated source. The higher light level should result in reduction of the standard uncertainty component associated with low-light transmittance measurements.

Several evaluation elements for the CCD camera system are reported below, in comparison to the scanning densitometer, where appropriate. To avoid confusion, the following nomenclature is employed across all of the discussions below. A *test region* of 5 mm by 20 mm is examined on each filter by each instrument, by sampling *test areas* of approximately 1 mm by 4 mm. The scanning densitometer, characterized as *XY* in tables and figures, determines the relative transmittance for nine such test areas using a single measurement for each, and the camera, characterized as *CCD* in figures and tables determines the relative transmittance for 25 test areas, using the average of 36 super pixels. This study is not concerned with total uncertainties for the measured transmittance, but only the component of uncertainty attributable to filter inhomogeneity for 1 mm by 4 mm sampled areas, a “systematic” effect, and that attributable to random effects (which degrades the ability of the instrument to distinguish inhomogeneity). Both of these uncertainty components are reported as *relative standard uncertainties*, which are computed as relative standard deviations. The systematic effect due to inhomogeneity is characterized by an *among-area* computation, utilizing the various test area transmittances, and the uncertainty arising from random sources is characterized using a *within-area* computation, employing super pixels and/or replication. Also reported with both instruments are *relative deviations*, which are differences between the area transmittances and the average transmittance for the region, divided by the latter. Both the relative deviations and the relative standard uncertainties are given (as noted in tables and figures) as percentages.

### 3.1 Images

In spite of the obvious variation in grey level, the eight filters shown in Fig. 2 are all nominal *T* = 20 % filters. The grey scale of the computer monitor is superior to that of the printer, but in either case the number of grey levels is limited to 8 bits (or one part in 256) of the 15 bit effective depth of the data. Thus, to enhance the contrast sufficiently to actually visually perceive small changes in transmittance, the maximum limits of greyscale are based upon the transmittance extremes for a particular filter under examination. This is a simple mouse-driven “Autoscale” operation in the software furnished with the camera. In Fig. 2, the greyscale has been “autoscaled” to position 1.

Some of the other filters in the holder may be appropriately scaled, but others may exceed the maximum “autoscaled” transmittance and appear totally white, and others may transmit less than the scaled minimum and appear totally black in the image. Average transmittances at 546.1 nm, as determined by the CCD camera, are listed in Table 1 for the eight nominal *T* = 20 % filters shown in Fig. 2.

Filters have been found to exhibit inhomogeneities of several types: 1) simple shading side-to-side, top-to-bottom, or on a bias; 2) irregular shading; and 3) point defects. The “planar” shading of the first type could arise from non-parallel faces of the filter or from a gradation in the absorptivity of the base glass. The irregular shading would most likely arise from variable absorptivity in the base glass. The point defects could arise from entrained particles or bubbles in the base glass, or from polishing grit embedded in the surface.

Apparent “point defects” which are indistinguishable from those appearing as black dots in Fig. 2 and discussed above have also arisen from dust on the filters. Care must be taken to assure that filters are not rejected for surface dust. Cleaning of the filters by firm wiping with optical tissue may result in static charging and the attraction of dust. A small alpha particle source (designed to neutralize statically charged samples before weighing) is brought near the filters for a minute before mounting in the instrument, and a rubber bulb is used to blow dust from the surface.

### 3.2 Position-by-Position Transmittance Correlation between the Two Instruments

A printout of a typical “report” file for the CCD camera system is shown in Table 2. Three values are given for each of the 25 test areas. The nominal transmittance at each position is the average over an area three super pixels wide by 12 super pixels high (along the long axis) for the 2 × 2 binned data, corresponding to the nominal 1 mm by 4 mm test area, as noted earlier and illustrated in Fig. 3. The second value given is the relative deviation between the measured transmittance for the position and the average transmittance for all 25 positions, and is a measure of inhomogeneity at the given position. The third value given for each position is the relative standard uncertainty (computed as a relative standard deviation) of the mean of the transmittance as determined from the 36 super pixels. This uncertainty may result from a combination of statistical photon counting fluctuations and small scale inhomogeneity on the scale of the 1 mm by 4 mm sampled area.

The report is geometrically laid out in rows and columns corresponding to the measurement positions illustrated in Fig. 3, for ease of localizing an area to examine on the high resolution image of a filter. Averages of the reported values by row and column help to characterize trends and shadings of transmittance behavior in a filter, and “grand averages” of the three quantities are given at the intersection of the row and column averages, in the lower right hand corner. The grand average of the 25 relative standard uncertainties of the mean is somewhat useful to characterize the overall repeatability of the transmittance measurement at a single position (see Sec. 3.6). The grand average of the 25 relative differences becomes zero by definition.

The relative standard uncertainty of the transmittance over the tested region of the filter is computed as the standard deviation of the 25 test area transmittance values divided by the average transmittance over the region, and is displayed near the bottom of the report. An expanded uncertainty component due to transmittance inhomogeneity is also given at the very bottom of the report, using an expansion factor of *k* = 2.064, defining a level of confidence close to 95 % for 24 degrees of freedom (see Sec. 3.3).

**Fig. 4:**
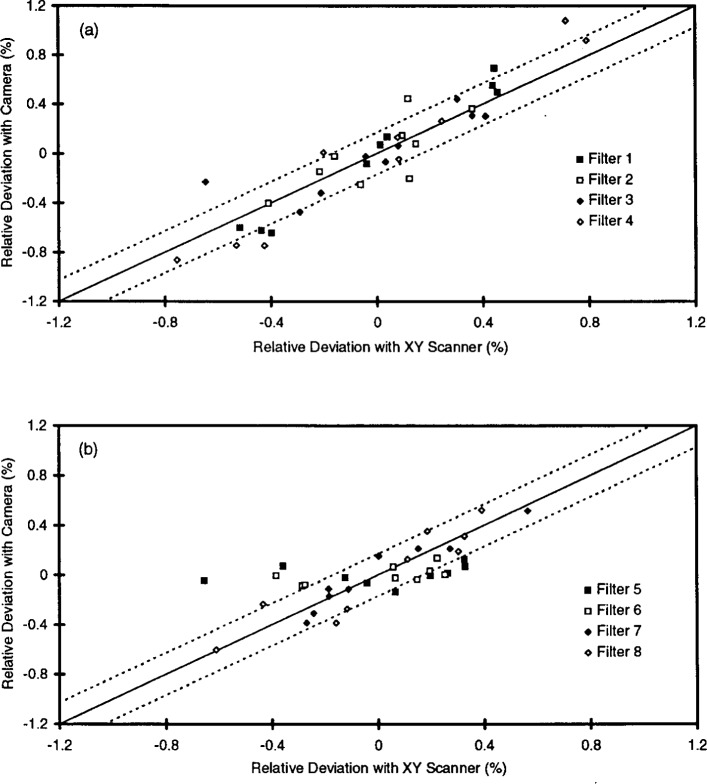
Correlation of relative transmittance deviations (from the average value for a filter) for nine positions on the eight filters of Fig. 2, as determined by the CCD camera (ordinate) and the scanning densitometer (abcissa). The data are arbitrarily separated into two plots for clarity.

Figure 4 shows the correlation between the camera and the scanning instrument for the nominal *T* = 20 % filters of Fig. 2 and Table 1. For each filter, and for each of the nine positions of the scanning densitometer, the relative deviation from the average transmittance as measured by the camera (at the same nominal position) is plotted as a function of the same quantity as measured by the scanning instrument. As mentioned earlier, each “position” represents a 1 mm wide by 4 mm high test area of the filter surface, with nine of the 25 positions reported for the camera data corresponding approximately to the nine positions reported by the scanning densitometer, as illustrated by the shaded areas in Fig. 3.

Perfect agreement, as represented by the solid diagonal line, would not be expected due to both statistical repeatability and the inexactitude of the spatial registration and scaling between the two systems. The dashed lines represent an expanded (*k* = 2) uncertainty for the statistical repeatability of both instruments, determined in the manner discussed in Sec. 3.4 below for one of the eight filters (number 4). The fact that more than 5 % of the data fall outside of the interval represents the imperfect spatial registration, in addition to questionable results for filter 5 on the scanning densitometer. A pair of later runs of filter 5 in the scanning densitometer failed to reproduce the wide transmittance density excursions indicated by Fig. 3. Regardless, the data of Fig. 4 serves as a “blunder check” for instrument artifacts, indicating that the two independent instruments detect corresponding transmittance deviations in real filter samples.

### 3.3 Test Area Transmittance Distribution Width

The distribution function for test area transmittances within a filter is not known, and may be different for the different sources of inhomogeneity discussed above. However, it is advantageous to compute the relative standard uncertainty for the nine transmittances determined by the scanning densitometer and the 25 determined by the camera system, for a given filter. This quantity yields a single measure of inhomogeneity (as opposed to the nine individual common positions), which should show correspondence between the two instruments.

Table 1 shows the transmittance relative standard uncertainties among test areas for the two instruments and the eight nominal *T* = 20 % filters of Figs. 2 and 4. For the CCD camera system, the results of three different measurements are given. The first two are back-to-back replications, without removal and replacement, to indicate the measurement variability resulting from photon statistics alone. Overall, the results agree with Fig. 4 in supporting general correspondence between the two instruments, with filters 5 and 6 providing the poorest agreement.

### 3.4 Filter Position in Holder

**Fig. 5:**
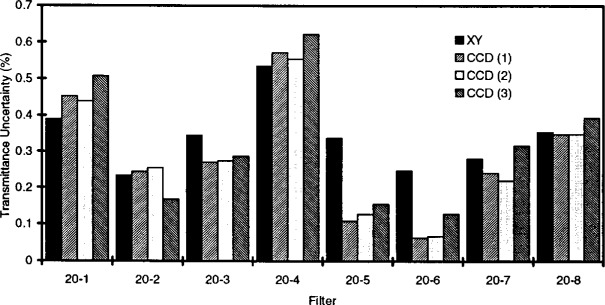
Bar chart comparing four different transmittance distribution width measurements (see Table 1) made for eight nominal *T* = 20 % filters: *XY*, percent relative standard uncertainty in transmittance of nine test areas using the scanning densitometer; CCD (1), percent relative standard uncertainty in transmittance of 25 test areas using the CCD camera system; CCD (2), simple replicate of CCD (1) without removal and replacement; CCD (3), replicate of CCD (1) and CCD (2), but following removal and replacement with a 180° rotation of the filter holder.

The final column of Table 1 represents a third replication of the camera data of the previous two columns, but after a 180° rotation of the filter holder. The filters are ordered in the table by their identity, not their position number. Figure 5 is a bar graph of all of the transmittance relative standard uncertainty values given in Table 1. The agreement of the camera data before and after filter holder rotation may be seen to be roughly equivalent to the agreement between the camera data and the scanning densitometer, and both agreements are poorer than the comparison of the back-to-back runs of the CCD camera with no filter interchange or movement. Because the magnification used was not chosen to yield an integral number of super pixels per filter position, the spatial registration is inexact, and the results do not match the other two runs as well as the other two runs match each other.

All eight filters of Table 1 and Figs. 2 and 4 were rejected as potential SRM filters on the basis of inhomogeneity as determined by the scanning instrument. The acceptance tolerance for relative transmittance inhomogeneity is 0.3 %, for an assumed *uniform* distribution, corresponding to an estimated relative standard uncertainty of about $0.3\;\%/\sqrt{3}=0.17\;\%$ for a normal distribution. Examination of Table 1 (as well as Fig. 4b) shows that filters 20-5 and 20-6 would have been acceptable as judged by the camera.

### 3.5 Performance Over Desired Transmittance Range

**Fig. 6:**
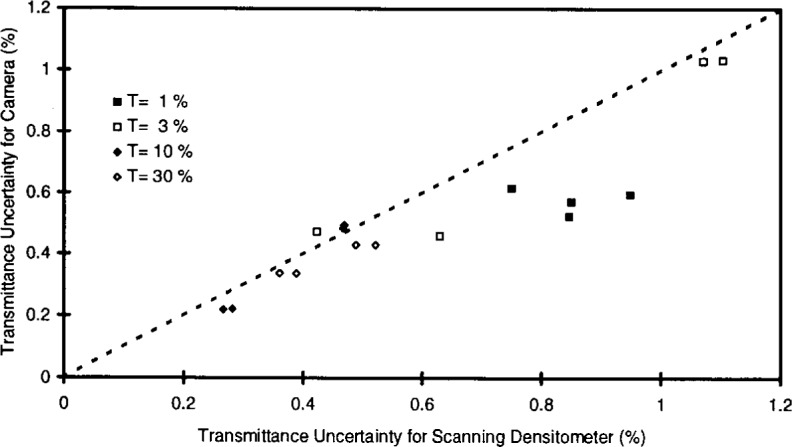
Correlation of the camera-based and densitometer-based determinations of inhomogeneity for measurements of two filters at each of four nominal transmittances, as given in the legend. The measure used for inhomogeneity is the relative standard uncertainty of test area transmittances for each filter, with 25 measurements for the camera and nine for the scanning densitometer.

The camera system will be required to determine transmittance homogeneity for filters whose nominal transmittance ranges between 1 % and 100 %. Figure 6 shows relative standard uncertainty values for the transmittance variation among test areas within a filter using the camera system, plotted as a function of the same quantity for the scanning system. Each data point of the figure represents a single run for a given filter, with two runs for each filter on both instruments, and two filters at each of four transmittance levels (*T*_nom_ = 1%, 3 %, 10 %, and 30 %), as indicated in the figure legend. The data for Fig. 6 are also reported in Table 3.

There is, of course, no particular relationship expected between the nominal transmittance of a filter and the variability of transmittance of the filter. This is especially evident for the two different *T* = 3 % filters, with a factor of about two between the indicated inhomogeneities. The correspondence between the instruments is quite good for transmittances down to 3 %, and somewhat less convincing at *T* = 1 %. In all cases, the replicate measurement reproduces better with the camera than with the densitometer.

This behavior arises from the fact that the relative standard uncertainty due to transmittance variability among test areas is confounded with an uncertainty due to random effects (such as photon detection noise, discussed below) which accounts for the systematic disagreement between the two instruments for both filters and runs at *T* = 1 % in Fig. 6, as well as accounting for the replication spread (when the filters are re-run without being re-positioned). The values plotted in Fig. 6 are inflated along *both* axes by the presence of random photon shot noise in addition to the desired true measure of transmittance variability. The apparent bias to the right of “ideal” correspondence indicated by the dashed line in the figure suggests that the random noise component is greater for the scanning densitometer than for the CCD camera system, as does the replication spread.

### 3.6 Within-Test-Area Statistical Repeatability

At all transmittances, but especially at lower ones, the measured transmittance at a given test area contains a component of uncertainty arising from random effects (e.g., photon statistics), which degrades the correlations of Figs. 4 and 6, and also may lead to the rejection of good filters (false negatives) or the acceptance of bad ones (false positives).

For the scanning densitometer, a single “run” of a filter contains no information to evaluate the uncertainty component resulting from random events in a reported transmittance deviation. In practice, a filter that “fails” because a relative deviation at a particular position exceeded the rejection threshold, is often rerun, maybe several times, to verify the consistency of the reading.

For the camera based instrument, the transmittance for each of the 25 test areas on a filter is computed using the average of 36 super pixels, so that the uncertainty in this quantity may be computed as the estimated standard deviation of the mean of the 36 super pixels. Of course, the uncertainty is only of random origin to the extent that the transmittance is homogeneous *within* a test area. Point defects, as described above, inflate this statistic above the estimated relative standard deviation of the average transmittance that would be found for repeated in-place measurements, and will probably be found in future studies to be recognizable as outliers to a normal probability distribution. Within a filter, the 25 estimated uncertainties should agree fairly well in the absence of such point defects. The average of these 25 uncertainties, reported as the last of the three “grand averages” in the lower right corner of the report, as shown in Table 2, should be a reasonable overall estimate of the relative uncertainty component resulting from random photon-counting statistics, assuming that a small number of the 25 test areas contain point defects.

**Fig. 7:**
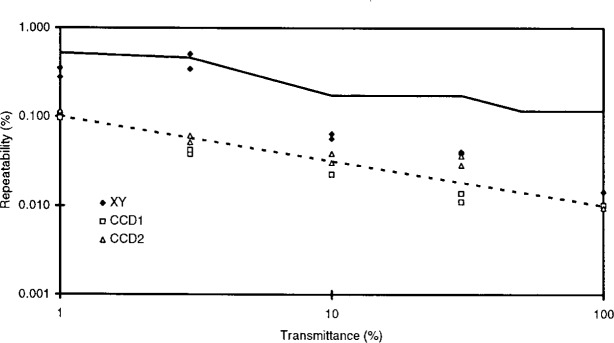
Comparative measures of statistical repeatability (arising from random effects) for the camera and scanner systems (from Table 3). The within-test-area estimated standard uncertainty was determined by a two-way analysis of variance with two-fold replication for the scanning densitometer (XY) and the CCD camera system (CCD1). An approximate estimate of the same statistic is given by the average of 25 within-test-area standard uncertainties of the mean of the transmittance for the camera system (CCD2). Each of these uncertainties is the relative standard deviation of the mean of the 36 pixels which are averaged for a single test area transmittance measurement. The dashed line indicates the functional behavior expected for photon “shot noise,” and the segmented line represents the homogeneity tolerances used for optical filter SRMs.

The metric discussed above is taken as one overall measure of repeatability for a transmittance level, and typical values are given in Table 3 under the heading of “CCD2” for two “runs” of two filters at each of the four transmittance levels, as well as for *T*_nom_ = 100 % (no filter in place in the filter holder). The results for “run 1” from Table 3 are plotted in Fig. 7, which compares several measures of limiting repeatability for the two instruments.

Also plotted in the figure is another measure which would be expected to report the same uncertainty (in the absence of point defects), and is labeled “CCD1” in both Fig. 7 and Table 3. For two back-to-back replicate runs on the camera system, the “position” data from the “reports” is subjected to a two-way Analysis of Variance (ANOVA) with replication (2-fold), with the 5 rows taken as one factor and the five columns as the second. The pairs of runs in Table 3 represent the 2-fold replication for the ANOVA, which reports a “within-test-area” variance based on 25 degrees of freedom. The square root of this variance, converted to relative percent, is also plotted in Fig. 7, and agrees closely with the “CCD2” metric at lower transmittances, diverging at higher transmittances (other than 100 %). The divergence may result from point defects having greater relative effect at higher transmittances.

The *T* = 100 % data plotted in Fig. 7 and shown in Table 3 was for two positions (4 and 8) left open for a single run. The repeatability was computed as for the other data, but the ANOVA treated the two openings as the two “replicates” unlike the filter data.

The third series plotted in Fig. 7 was computed using a two-way ANOVA with two-fold replication for the scanning densitometer, and is indicated in the figure and in Table 3 as “XY.” The factors were the three rows and the three columns measured in the densitometer. The square root of the “within” position variance, expressed as a percent relative standard uncertainty, is seen to exceed the corresponding measure for the camera by a factor of about 5, over the entire dynamic range. The *T* = 100 % data for the scanning densitometer was computed in the same way as for the filters, using two separate runs with no filter in place.

For reference, Fig. 7 contains a representation (unlabeled dashed line) of the theoretical dependence of photon “shot” noise on transmitted intensity, with arbitrary intercept. The log-log slope of −1/2 (one decade decrease in relative uncertainty per two decade increase in transmitted intensity) would be expected to be characteristic of random uncertainty associated with photon detection from a stable source. The segmented, solid curve in the figure represents the rejection thresholds used for the different transmittance levels, expressed as the relative standard uncertainty. False rejections and acceptances obviously are more frequent as the repeatability uncertainty component (attributed to random noise sources) approaches the decision threshold.

### 3.7 Background Light

**Fig. 8:**
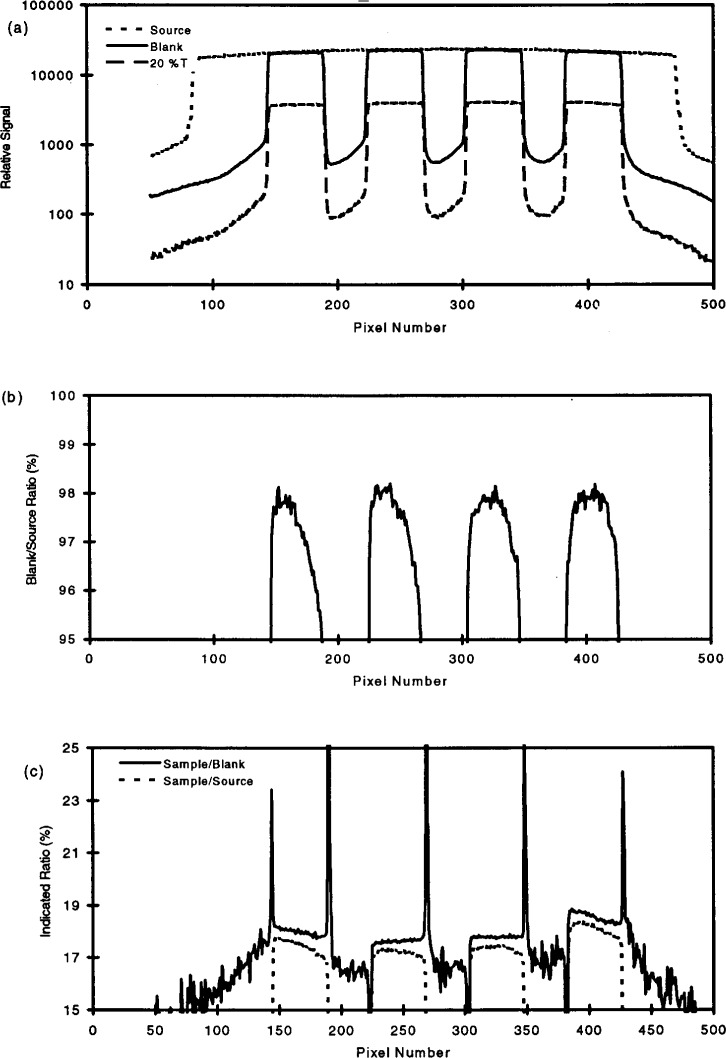
(a) Raw data from a single row of camera pixels (row 117), which crosses the center of filter positions 1–4, for the extended source alone (dotted curve), the extended source with the blank mask in place (solid curve), and the extended source with *T*_nom_ = 20 % filters in the filter holder (dashed curve). (b) The ratio (in percent) of the blank curve to the extended source curve of part a. (c) The ratios (in percent) of the sample curve to the source curve (dotted curve) and the sample curve to the blank curve (solid curve) of part a.

The curves shown in Fig. 8 are derived using the numerical data from a single row of pixels, for 1) extended light source with neither the blank nor samples in place; 2) the blank; and 3) the nominal *T* = 20 % filters of Fig. 2. The pixel row crosses filter positions 1 through 4 near the center of the filters. Figure 8a simply shows the raw data for the three conditions noted above. The logarithmic ordinate emphasizes the presence of unwanted light in regions which should be “dark.” Of course, the dark areas are of no interest directly, but the indicated behavior suggests the influence of adjacent filter positions on each other. Comparing the “blank” and “*T*_nom_ = 20 %” curves in Fig. 8a indicates that the unwanted background scales linearly with nominal filter transmittance, resulting in a constant offset on the logarithmic ordinate of the plot. Thus the potential influence of “spill” light from adjacent filter positions ratios out in the flatfield correction, accounting for the ability of the system to diagnose transmittance differences at the level of 0.1 % relative standard uncertainty while experiencing a light background above 1 % of the reference light level.

Figure 8b emphasizes the importance of using the blank for flatfield correction (instead of the full source), by showing a plot of the ratio of the blank to the source for the row of pixels. The blank can be seen to result in an apparent reduction of about 2 % for the incident light level through the four clear apertures, and the transmitted light relative to the source is not even uniform across each opening. It is not clear to what extent this effect and the dark background effect is optical (diffraction and scattering) or pixel-to-pixel electronic crosstalk in the CCD array.

Figure 8c compares normalization of the filter data to the source (broken line) or to the blank (solid line). Obviously, due to the blank effect indicated in Fig. 8b, the predictions of the two normalizations differ both for transmittance and uniformity. The congruence of the filter and blank curves and the agreement with the scanning densitometer support the use of the blank for normalization. The positive and negative spikes shown for blank normalization represent the effect of mechanical tolerances for positioning and hole size, between the blank and the filter holder.

The data of Fig. 8 suggest the following operational procedures:

1. The “flatfield” file must be taken with the blank in place (not an unapertured view of the light source);
2. All filter positions within a few millimeters of each other should be occupied by filters of the same nominal transmittance; and,
3. Filters of different nominal transmittance should be separated by one or more positions which are “masked” off in both the flatfield and sample exposures.

## 4. Conclusions

This study has shown a consistent congruence between the results of a new instrument for evaluating optical filter uniformity and an instrument which has performed the function for the past decade. The study used only filters which the older instrument had shown to be unacceptably inhomogeneous for use as NIST SRMs. The operational limit not evident in the samples employed is the agreement of the instruments for *homogeneous* filters. This behavior is actually explored indirectly in the study of statistical repeatability, which cannot be distinguished from inhomogeneity in the homogeneous limit. The only truly uniform “sample” available, *T* = 100 % (no filter in place), yielded consistent results with the real filters in the repeatability studies, which were designed to isolate the component of uncertainty attributable to random events such as photon counting statistics.

These studies indicate the minimum values of transmittance variation detectable on the two systems, and show the performance of the CCD camera to be superior to that of the scanning instrument for the exposure times used. Of course, the relative uncertainty arising from random events for either instrument may be improved by lengthening the signal averaging time. Although *significant* improvement would involve unacceptable measurement times for either instrument, it may be worth lengthening the camera exposure time somewhat for the lower transmittance filters, since the acquisition time is dominated by computer processing, not by the actual exposure time.

The following observations may be applied to the new CCD camera system:

1. The camera system yields sufficient results-conformity with the scanning densitometer to take over the functions of the latter.
2. The new system improves throughput by examining multiple filters in parallel.
3. Parallel examination of all areas of a filter reduces the importance of source stability, permitting the use of a more intense source with concommitant improvement in the statistical measurement repeatability.
4. The numerical, statistical treatment of high spatial resolution data to determine millimeter scale homogeneity results in an uncertainty estimate for each measured transmittance deviation, a useful feature (not present in the older instrument) for distinguishing sample homogeneity from measurement repeatability.
5. Visual observation and analysis of contrast-enhanced high resolution images may help improve the production quality of optical filter standard reference materials.

The results of this study qualify the CCD camera system to replace the scanning densitometer for the evaluation of the uniformity of optical filter standard reference materials. Further, the camera images will be used in consultation with the bulk material suppliers and with the NIST optical shops to raise the production quality and lower the rejection rate of these SRMs.

## Figures and Tables

**Table 1:** Comparative uniformity for eight nominal *T* = 20 % filters

Filter	Average transmittance		(Transmittance relative standard uncertainty)/%^a^		
		XY^b^	CCD^c^ (1)	CCD^c^ (2^d^)	CCD^c^ (3^e^)
20-1	0.182	0.390	0.452	0.439	0.507
20-2	0.179	0.234	0.244	0.255	0.168
20-3	0.180	0.344	0.270	0.274	0.287
20-4	0.188	0.534	0.571	0.553	0.621
20-5	0.178	0.337	0.108	0.127	0.155
20-6	0.180	0.247	0.063	0.067	0.128
20-7	0.173	0.280	0.241	0.219	0.316
20-8	0.180	0.354	0.348	0.348	0.393

a The estimated relative standard uncertainty of *N* (see below) transmittances determined for 1 mm by 4 mm subregions covering a 5 mm by 20 mm region of the face of the filter.

b Determined using the scanning densitometer (*N* = 9).

c Determined using the CCD camera system (*N* = 25).

d Simple repeat of the CCD run, without removal and replacement of samples.

e CCD run with sample holder turned 180°, such that filter 1 occupies position 8, etc.

**Table 2:** Report of transmittance uniformity for filter 20-1

17 augal.rpt						
Within Each Sub-area:						
Transmittance						
Relative Difference from Average Transmittance/%						
Relative Standard Uncertainty of Transmittance/%						
	1	2	3	4	5	Row Avg.
1	0.1828	0.1825	0.1821	0.1816	0.1808	0.1820
1	0.4841	0.2847	0.0560	−0.2110	−0.6160	−0.0004
1	0.0196	0.0204	0.0224	0.0224	0.0303	0.0230
2	0.1829	0.1825	0.1821	0.1815	0.1808	0.1820
2	0.5312	0.2697	0.0588	−0.2310	−0.6494	−0.0041
2	0.0288	0.0246	0.0254	0.0286	0.0258	0.0267
3	0.1829	0.1826	0.1822	0.1814	0.1808	0.1820
3	0.5404	0.3612	0.1220	−0.2977	−0.6581	0.0135
3	0.0531	0.0221	0.0249	0.0273	0.0272	0.0309
4	0.1832	0.1825	0.1821	0.1814	0.1807	0.1820
4	0.6943	0.2928	0.0833	−0.3339	−0.7115	0.0050
4	0.0230	0.0762	0.0317	0.0257	0.0294	0.0372
5	0.1832	0.1826	0.1818	0.1813	0.1808	0.1819
5	0.6805	0.3635	−0.0971	−0.3806	−0.6372	−0.0142
5	0.0262	0.0236	0.0338	0.0250	0.0270	0.0271
ColAv	0.1830	0.1825	0.1820	0.1814	0.1808	0.1820
ColAv	0.5861	0.3144	0.0446	−0.2908	−0.6545	−0.0000
ColAv	0.0302	0.0334	0.0277	0.0258	0.0279	0.0290
Among Sub-areas:						
Average Transmittance is.1819623						
Relative Standard Uncertainty of Transmittance is 0.4522 %						
Relative Expanded Uncertainty of Transmittance is 0.9333 %						

**Table 3:** Summary data for two filters at each of four levels and *T* = 100 %

Filter	*T*_nom_/%^a^	Run	(Transmittance rel. std. unc.)/%^b^			Repeatability/%^e^	
			XY^c^	CCD^d^	XY^f^	CCD1^g^	CCD2^h^
1-1	1	1	0.847	0.523	0.276	0.106	0.111
		2	0.851	0.571			0.108
1-2		1	0.750	0.615	0.351	0.096	0.115
		2	0.949	0.594			0.110
3-1	3	1	1.104	1.033	0.344	0.038	0.061
		2	1.071	1.028			0.062
3-2		1	0.630	0.459	0.506	0.042	0.052
		2	0.423	0.473			0.051
10-1	10	1	0.470	0.494	0.064	0.022	0.039
		2	0.467	0.484			0.038
10-2		1	0.282	0.221	0.056	0.022	0.030
		2	0.266	0.218			0.031
30-1	30	1	0.522	0.430	0.038	0.014	0.029
		2	0.489	0.430			0.029
30-2		1	0.361	0.337	0.040	0.011	0.036
		2	0.389	0.337			0.036
N.A.	100	1	N.A.	N.A.	0.014	0.010	0.010
		2					0.010

a Nominal transmittance, expressed as a percentage.

b The estimated relative standard uncertainty of *N* (see below) transmittances determined for 1 mm by 4 mm subregions covering a 5 mm by 20 mm region of the face of the filter.

c Determined using the scanning densitometer (*N* = 9).

d Determined using the CCD camera system (*N* = 25).

e The relative standard deviation of the mean transmittance for a single test area of a filter.

f Determined using two-way ANOVA with replication (two runs shown) for three horizontal and three vertical positions of the scanning densitometer.

g Determined using two-way ANOVA with replication (two runs shown) for five “horizontal” and five “vertical” positions (as displayed) of the CCD camera system.

h The average of the computed relative standard deviations of the mean transmittance at each of 25 positions using the CCD camera system. Each of the 25 standard uncertainties is the computed with 36 super pixels whose average yields the sub-region transmittance.

## Footnotes

1 Certain commercial equipment, instruments, or materials are identified in this paper to foster understanding. Such identification does not imply recommendation or endorsement by the National Institute of Standards and Technology, nor does it imply that the materials or equipment identified are necessarily the best available for the purpose.

2 The effect of accumulated charge overflowing the well capacity of a given pixel and spilling into adjacent pixels.
